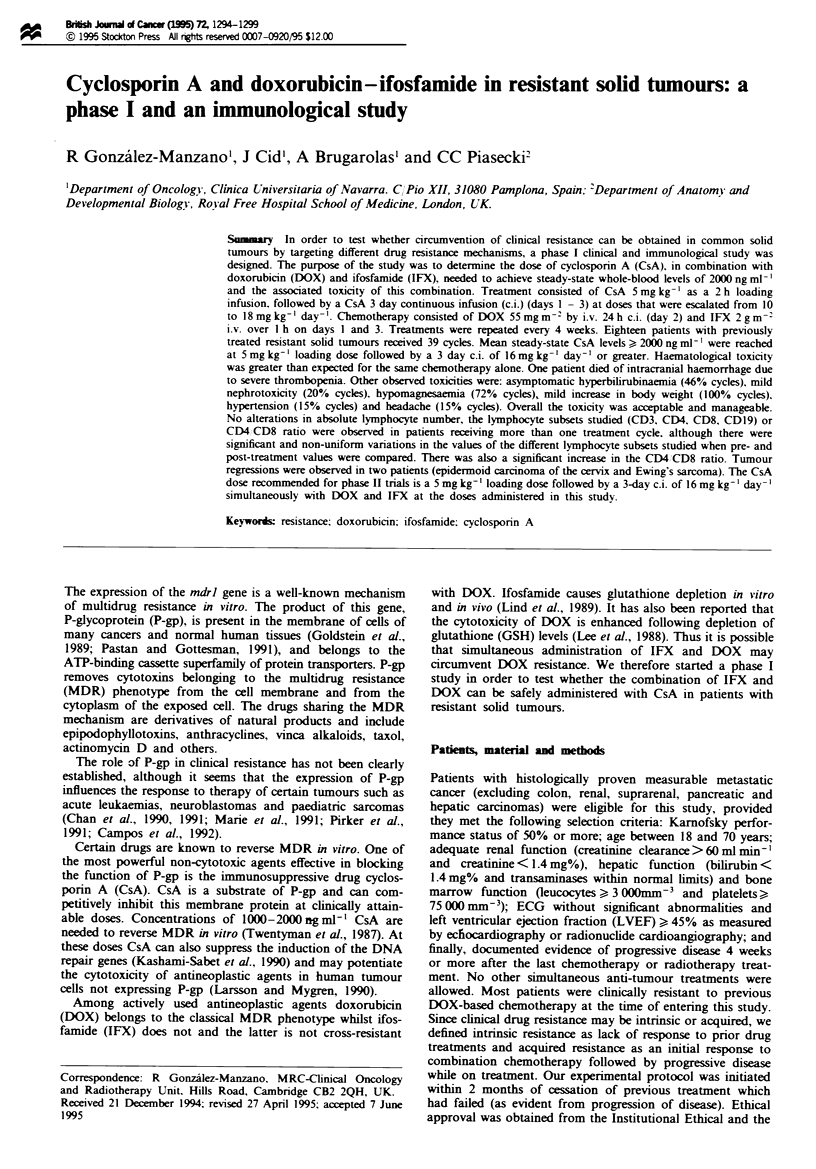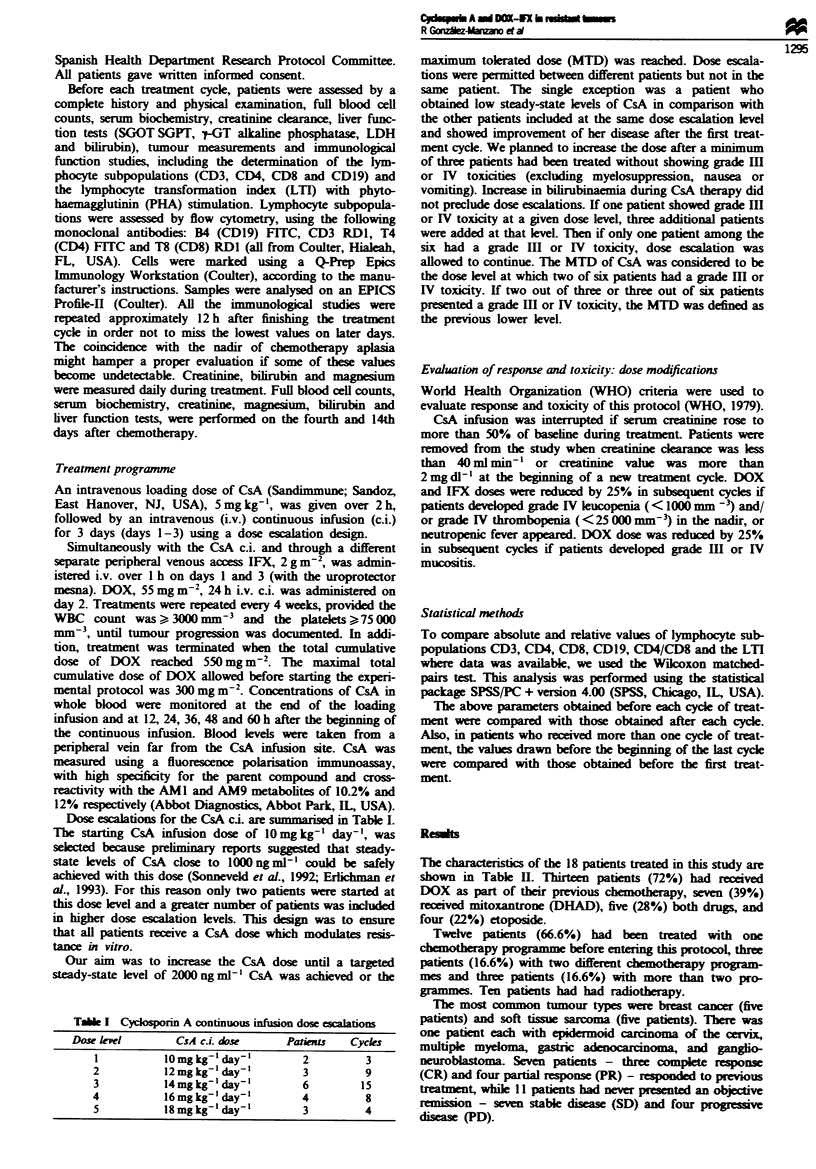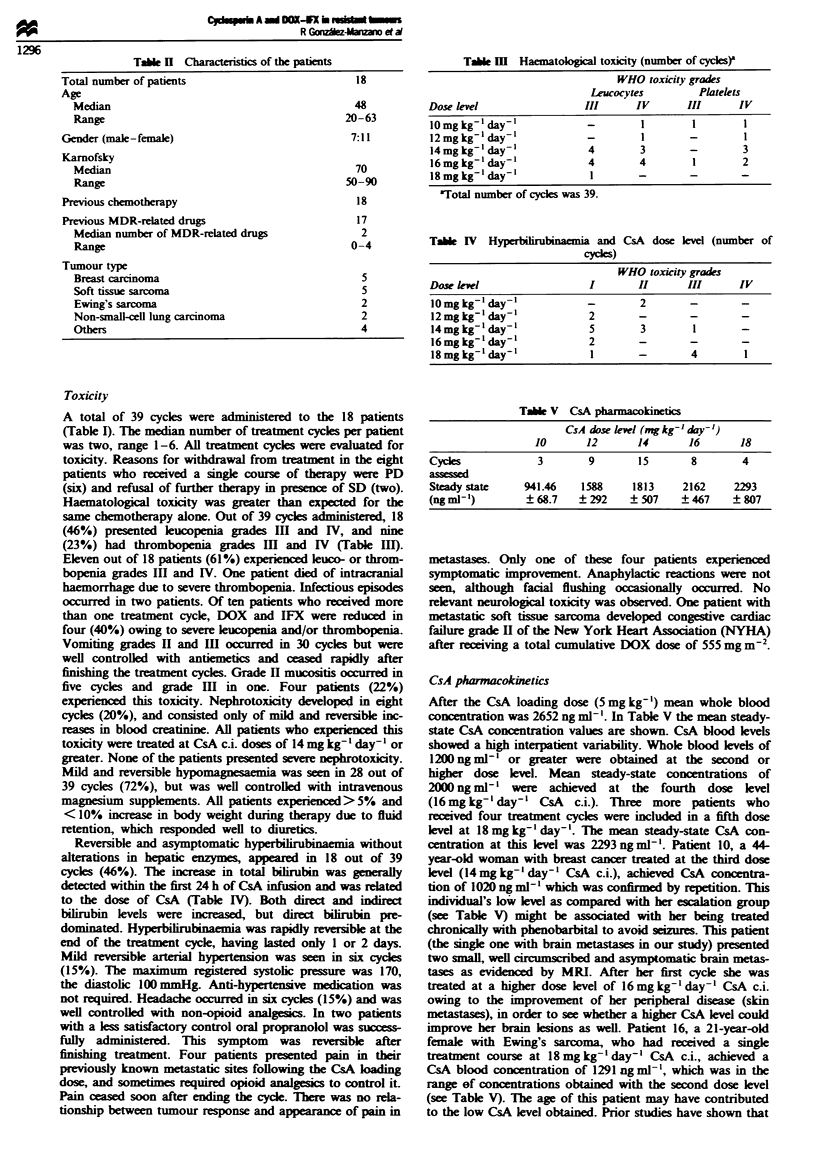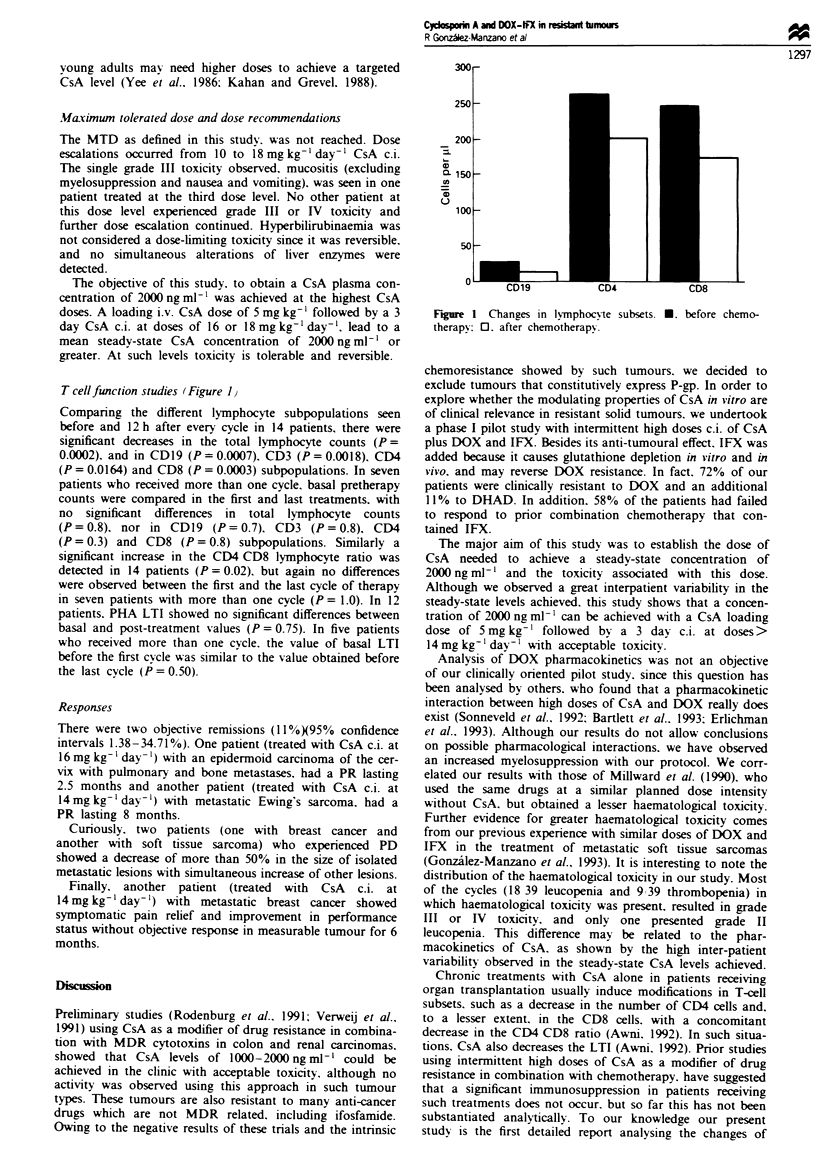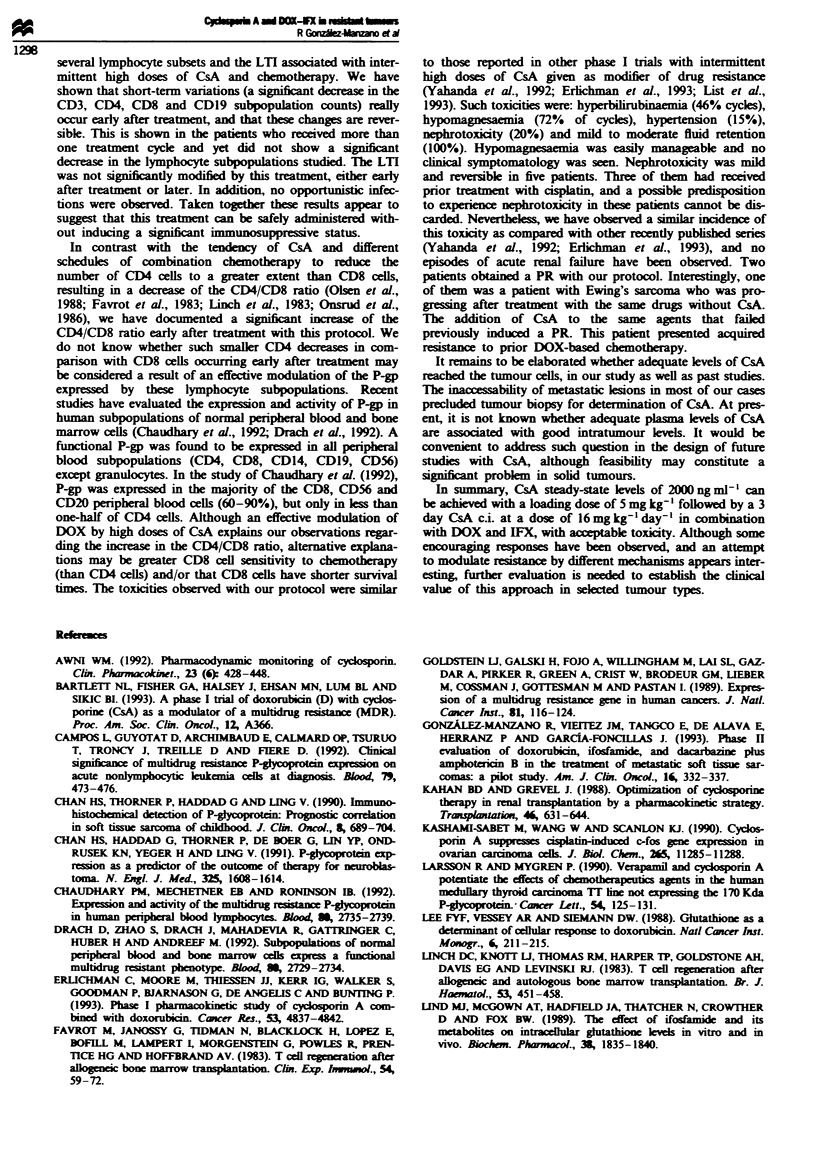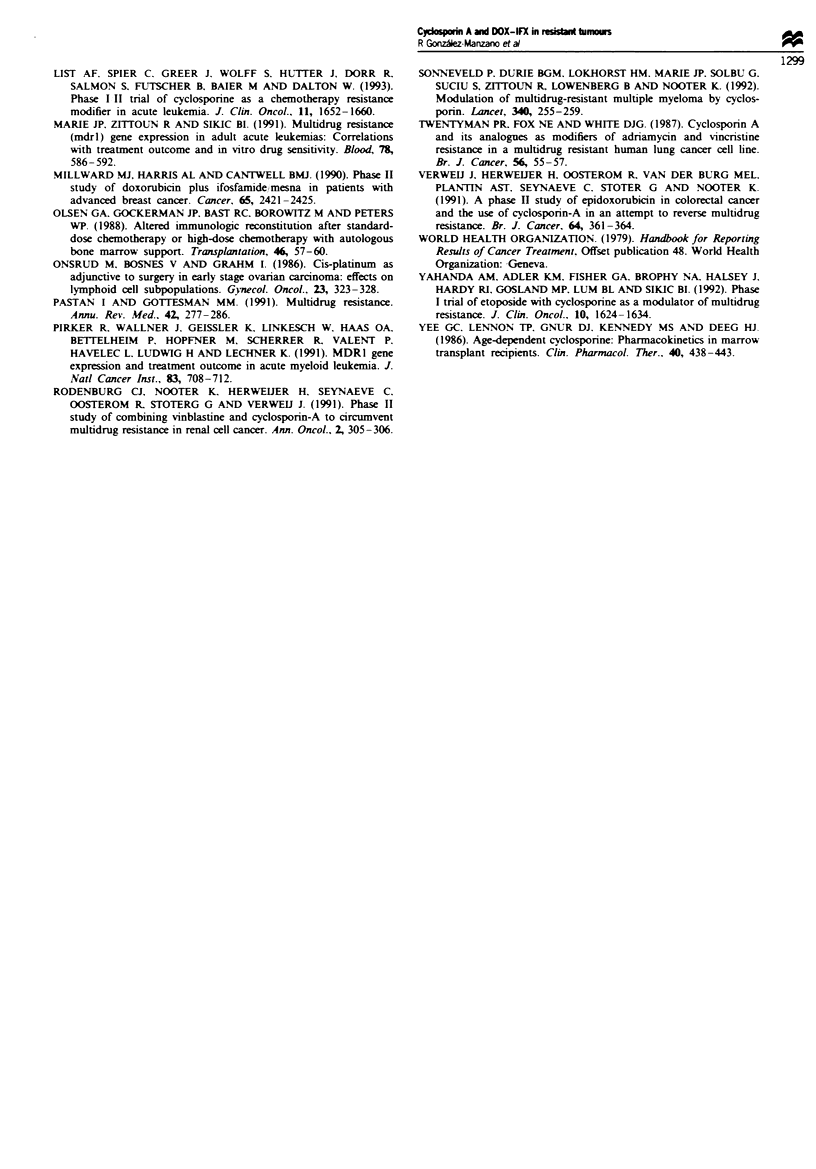# Cyclosporin A and doxorubicin-ifosfamide in resistant solid tumours: a phase I and an immunological study.

**DOI:** 10.1038/bjc.1995.503

**Published:** 1995-11

**Authors:** R. González-Manzano, J. Cid, A. Brugarolas, C. C. Piasecki

**Affiliations:** Department of Oncology, Clinica Universitaria of Navarra, Pamplona, Spain.

## Abstract

In order to test whether circumvention of clinical resistance can be obtained in common solid tumours by targeting different drug resistance mechanisms, a phase I clinical and immunological study was designed. The purpose of the study was to determine the dose of cyclosporin A (CsA), in combination with doxorubicin (DOX) and ifosfamide (IFX), needed to achieve steady-state whole-blood levels of 2000 ng ml-1 and the associated toxicity of this combination. Treatment consisted of CsA 5 mg kg-1 as a 2 h loading infusion, followed by a CsA 3 day continuous infusion (c.i.) (days 1-3) at doses that were escalated from 10 to 18 mg kg-1 day-1. Chemotherapy consisted of DOX 55 mg m-2 by i.v. 24 h c.i. (day 2) and IFX 2 g m-2 i.v. over 1 h on days 1 and 3. Treatments were repeated every 4 weeks. Eighteen patients with previously treated resistant solid tumours received 39 cycles. Mean steady-state CsA levels > or = 2000 ng ml-1 were reached at 5 mg kg-1 loading dose followed by a 3 day c.i. of 16 mg kg-1 day-1 or greater. Haematological toxicity was greater than expected for the same chemotherapy alone. One patient died of intracranial haemorrhage due to severe thrombopenia. Other observed toxicities were: asymptomatic hyperbilirubinaemia (46% cycles), mild nephrotoxicity (20% cycles), hypomagnesaemia (72% cycles), mild increase in body weight (100% cycles), hypertension (15% cycles) and headache (15% cycles). Overall the toxicity was acceptable and manageable. No alterations in absolute lymphocyte number, the lymphocyte subsets studied (CD3, CD4, CD8, CD19) or CD4/CD8 ratio were observed in patients receiving more than one treatment cycle, although there were significant and non-uniform variations in the values of the different lymphocyte subsets studied when pre- and post-treatment values were compared. There was also a significant increase in the CD4/CD8 ratio. Tumour regressions were observed in two patients (epidermoid carcinoma of the cervix and Ewing's sarcoma). The CsA dose recommended for phase II trials is a 5 mg kg-1 loading dose followed by a 3-day c.i. of 16 mg kg-1 day-1 simultaneously with DOX and IFX at the doses administered in this study.


British Journal of Cancer (1995) 72, 1294–1299

© 1995 Stockton Press All rights reserved 0007-0920/95 $12.00

### Cyclosporin A and doxorubicin–ifosfamide in resistant solid tumours: a phase I and an immunological study

R González-Manzano[1], J Cid[1], A Brugarolas[1] and CC Piasecki[2]

[1]*Department of Oncology, Clínica Universitaria of Navarra, C/Pio XII, 31080 Pamplona, Spain;* [2]*Department of Anatomy and Developmental Biology, Royal Free Hospital School of Medicine, London, UK.*

**Summary** In order to test whether circumvention of clinical resistance can be obtained in common solid tumours by targeting different drug resistance mechanisms, a phase I clinical and immunological study was designed. The purpose of the study was to determine the dose of cyclosporin A (CsA), in combination with doxorubicin (DOX) and ifosfamide (IFX), needed to achieve steady-state whole-blood levels of 2000 ng ml$^{-1}$ and the associated toxicity of this combination. Treatment consisted of CsA 5 mg kg$^{-1}$ as a 2 h loading infusion, followed by a CsA 3 day continuous infusion (c.i.) (days 1 – 3) at doses that were escalated from 10 to 18 mg kg$^{-1}$ day$^{-1}$. Chemotherapy consisted of DOX 55 mg m$^{-2}$ by i.v. 24 h c.i. (day 2) and IFX 2 g m$^{-2}$ i.v. over 1 h on days 1 and 3. Treatments were repeated every 4 weeks. Eighteen patients with previously treated resistant solid tumours received 39 cycles. Mean steady-state CsA levels $\geqslant$ 2000 ng ml$^{-1}$ were reached at 5 mg kg$^{-1}$ loading dose followed by a 3 day c.i. of 16 mg kg$^{-1}$ day$^{-1}$ or greater. Haematological toxicity was greater than expected for the same chemotherapy alone. One patient died of intracranial haemorrhage due to severe thrombopenia. Other observed toxicities were: asymptomatic hyperbilirubinaemia (46% cycles), mild nephrotoxicity (20% cycles), hypomagnesaemia (72% cycles), mild increase in body weight (100% cycles), hypertension (15% cycles) and headache (15% cycles). Overall the toxicity was acceptable and manageable. No alterations in absolute lymphocyte number, the lymphocyte subsets studied (CD3, CD4, CD8, CD19) or CD4/CD8 ratio were observed in patients receiving more than one treatment cycle, although there were significant and non-uniform variations in the values of the different lymphocyte subsets studied when pre- and post-treatment values were compared. There was also a significant increase in the CD4/CD8 ratio. Tumour regressions were observed in two patients (epidermoid carcinoma of the cervix and Ewing's sarcoma). The CsA dose recommended for phase II trials is a 5 mg kg$^{-1}$ loading dose followed by a 3-day c.i. of 16 mg kg$^{-1}$ day$^{-1}$ simultaneously with DOX and IFX at the doses administered in this study.

**Keywords:** resistance; doxorubicin; ifosfamide; cyclosporin A

The expression of the *mdr1* gene is a well-known mechanism of multidrug resistance *in vitro*. The product of this gene, P-glycoprotein (P-gp), is present in the membrane of cells of many cancers and normal human tissues (Goldstein *et al*., 1989; Pastan and Gottesman, 1991), and belongs to the ATP-binding cassette superfamily of protein transporters. P-gp removes cytotoxins belonging to the multidrug resistance (MDR) phenotype from the cell membrane and from the cytoplasm of the exposed cell. The drugs sharing the MDR mechanism are derivatives of natural products and include epipodophyllotoxins, anthracyclines, vinca alkaloids, taxol, actinomycin D and others.

The role of P-gp in clinical resistance has not been clearly established, although it seems that the expression of P-gp influences the response to therapy of certain tumours such as acute leukaemias, neuroblastomas and paediatric sarcomas (Chan *et al*., 1990, 1991; Marie *et al*., 1991; Pirker *et al*., 1991; Campos *et al*., 1992).

Certain drugs are known to reverse MDR *in vitro*. One of the most powerful non-cytotoxic agents effective in blocking the function of P-gp is the immunosuppressive drug cyclosporin A (CsA). CsA is a substrate of P-gp and can competitively inhibit this membrane protein at clinically attainable doses. Concentrations of 1000–2000 ng ml$^{-1}$ CsA are needed to reverse MDR *in vitro* (Twentyman *et al*., 1987). At these doses CsA can also suppress the induction of the DNA repair genes (Kashami-Sabet *et al*., 1990) and may potentiate the cytotoxicity of antineoplastic agents in human tumour cells not expressing P-gp (Larsson and Mygren, 1990).

Among actively used antineoplastic agents doxorubicin (DOX) belongs to the classical MDR phenotype whilst ifosfamide (IFX) does not and the latter is not cross-resistant with DOX. Ifosfamide causes glutathione depletion *in vitro* and *in vivo* (Lind *et al*., 1989). It has also been reported that the cytotoxicity of DOX is enhanced following depletion of glutathione (GSH) levels (Lee *et al*., 1988). Thus it is possible that simultaneous administration of IFX and DOX may circumvent DOX resistance. We therefore started a phase I study in order to test whether the combination of IFX and DOX can be safely administered with CsA in patients with resistant solid tumours.

### Patients, material and methods

Patients with histologically proven measurable metastatic cancer (excluding colon, renal, suprarenal, pancreatic and hepatic carcinomas) were eligible for this study, provided they met the following selection criteria: Karnofsky performance status of 50% or more; age between 18 and 70 years; adequate renal function (creatinine clearance > 60 ml min$^{-1}$ and creatinine < 1.4 mg%), hepatic function (bilirubin < 1.4 mg% and transaminases within normal limits) and bone marrow function (leucocytes $\geqslant$ 3 000mm$^{-3}$ and platelets $\geqslant$ 75 000 mm$^{-3}$); ECG without significant abnormalities and left ventricular ejection fraction (LVEF) $\geqslant$ 45% as measured by echocardiography or radionuclide cardioangiography; and finally, documented evidence of progressive disease 4 weeks or more after the last chemotherapy or radiotherapy treatment. No other simultaneous anti-tumour treatments were allowed. Most patients were clinically resistant to previous DOX-based chemotherapy at the time of entering this study. Since clinical drug resistance may be intrinsic or acquired, we defined intrinsic resistance as lack of response to prior drug treatments and acquired resistance as an initial response to combination chemotherapy followed by progressive disease while on treatment. Our experimental protocol was initiated within 2 months of cessation of previous treatment which had failed (as evident from progression of disease). Ethical approval was obtained from the Institutional Ethical and the

Correspondence: R González-Manzano, MRC-Clinical Oncology and Radiotherapy Unit, Hills Road, Cambridge CB2 2QH, UK.
Received 21 December 1994; revised 27 April 1995; accepted 7 June 1995

Spanish Health Department Research Protocol Committee. All patients gave written informed consent.

Before each treatment cycle, patients were assessed by a complete history and physical examination, full blood cell counts, serum biochemistry, creatinine clearance, liver function tests (SGOT SGPT, γ-GT alkaline phosphatase, LDH and bilirubin), tumour measurements and immunological function studies, including the determination of the lymphocyte subpopulations (CD3, CD4, CD8 and CD19) and the lymphocyte transformation index (LTI) with phytohaemagglutinin (PHA) stimulation. Lymphocyte subpopulations were assessed by flow cytometry, using the following monoclonal antibodies: B4 (CD19) FITC, CD3 RD1, T4 (CD4) FITC and T8 (CD8) RD1 (all from Coulter, Hialeah, FL, USA). Cells were marked using a Q-Prep Epics Immunology Workstation (Coulter), according to the manufacturer's instructions. Samples were analysed on an EPICS Profile-II (Coulter). All the immunological studies were repeated approximately 12 h after finishing the treatment cycle in order not to miss the lowest values on later days. The coincidence with the nadir of chemotherapy aplasia might hamper a proper evaluation if some of these values become undetectable. Creatinine, bilirubin and magnesium were measured daily during treatment. Full blood cell counts, serum biochemistry, creatinine, magnesium, bilirubin and liver function tests, were performed on the fourth and 14th days after chemotherapy.

*Treatment programme*

An intravenous loading dose of CsA (Sandimmune; Sandoz, East Hanover, NJ, USA), 5 mg $kg^{-1}$, was given over 2 h, followed by an intravenous (i.v.) continuous infusion (c.i.) for 3 days (days 1–3) using a dose escalation design.

Simultaneously with the CsA c.i. and through a different separate peripheral venous access IFX, 2 g $m^{-2}$, was administered i.v. over 1 h on days 1 and 3 (with the uroprotector mesna). DOX, 55 mg $m^{-2}$, 24 h i.v. c.i. was administered on day 2. Treatments were repeated every 4 weeks, provided the WBC count was $\geq$3000 $mm^{-3}$ and the platelets $\geq$75 000 $mm^{-3}$, until tumour progression was documented. In addition, treatment was terminated when the total cumulative dose of DOX reached 550 mg $m^{-2}$. The maximal total cumulative dose of DOX allowed before starting the experimental protocol was 300 mg $m^{-2}$. Concentrations of CsA in whole blood were monitored at the end of the loading infusion and at 12, 24, 36, 48 and 60 h after the beginning of the continuous infusion. Blood levels were taken from a peripheral vein far from the CsA infusion site. CsA was measured using a fluorescence polarisation immunoassay, with high specificity for the parent compound and cross-reactivity with the AM1 and AM9 metabolites of 10.2% and 12% respectively (Abbot Diagnostics, Abbot Park, IL, USA).

Dose escalations for the CsA c.i. are summarised in Table I. The starting CsA infusion dose of 10 mg $kg^{-1}$ $day^{-1}$, was selected because preliminary reports suggested that steady-state levels of CsA close to 1000 ng $ml^{-1}$ could be safely achieved with this dose (Sonneveld *et al.*, 1992; Erlichman *et al.*, 1993). For this reason only two patients were started at this dose level and a greater number of patients was included in higher dose escalation levels. This design was to ensure that all patients receive a CsA dose which modulates resistance *in vitro*.

Our aim was to increase the CsA dose until a targeted steady-state level of 2000 ng $ml^{-1}$ CsA was achieved or the maximum tolerated dose (MTD) was reached. Dose escalations were permitted between different patients but not in the same patient. The single exception was a patient who obtained low steady-state levels of CsA in comparison with the other patients included at the same dose escalation level and showed improvement of her disease after the first treatment cycle. We planned to increase the dose after a minimum of three patients had been treated without showing grade III or IV toxicities (excluding myelosuppression, nausea or vomiting). Increase in bilirubinaemia during CsA therapy did not preclude dose escalations. If one patient showed grade III or IV toxicity at a given dose level, three additional patients were added at that level. Then if only one patient among the six had a grade III or IV toxicity, dose escalation was allowed to continue. The MTD of CsA was considered to be the dose level at which two of six patients had a grade III or IV toxicity. If two out of three or three out of six patients presented a grade III or IV toxicity, the MTD was defined as the previous lower level.

**Table I** Cyclosporin A continuous infusion dose escalations

| *Dose level* | *CsA c.i. dose* | *Patients* | *Cycles* |
|---|---|---|---|
| 1 | 10 mg $kg^{-1}$ $day^{-1}$ | 2 | 3 |
| 2 | 12 mg $kg^{-1}$ $day^{-1}$ | 3 | 9 |
| 3 | 14 mg $kg^{-1}$ $day^{-1}$ | 6 | 15 |
| 4 | 16 mg $kg^{-1}$ $day^{-1}$ | 4 | 8 |
| 5 | 18 mg $kg^{-1}$ $day^{-1}$ | 3 | 4 |

*Evaluation of response and toxicity: dose modifications*

World Health Organization (WHO) criteria were used to evaluate response and toxicity of this protocol (WHO, 1979).

CsA infusion was interrupted if serum creatinine rose to more than 50% of baseline during treatment. Patients were removed from the study when creatinine clearance was less than 40 ml $min^{-1}$ or creatinine value was more than 2 mg $dl^{-1}$ at the beginning of a new treatment cycle. DOX and IFX doses were reduced by 25% in subsequent cycles if patients developed grade IV leucopenia (<1000 $mm^{-3}$) and/or grade IV thrombopenia (<25 000 $mm^{-3}$) in the nadir, or neutropenic fever appeared. DOX dose was reduced by 25% in subsequent cycles if patients developed grade III or IV mucositis.

*Statistical methods*

To compare absolute and relative values of lymphocyte subpopulations CD3, CD4, CD8, CD19, CD4/CD8 and the LTI where data was available, we used the Wilcoxon matched-pairs test. This analysis was performed using the statistical package SPSS/PC + version 4.00 (SPSS, Chicago, IL, USA).

The above parameters obtained before each cycle of treatment were compared with those obtained after each cycle. Also, in patients who received more than one cycle of treatment, the values drawn before the beginning of the last cycle were compared with those obtained before the first treatment.

### Results

The characteristics of the 18 patients treated in this study are shown in Table II. Thirteen patients (72%) had received DOX as part of their previous chemotherapy, seven (39%) received mitoxantrone (DHAD), five (28%) both drugs, and four (22%) etoposide.

Twelve patients (66.6%) had been treated with one chemotherapy programme before entering this protocol, three patients (16.6%) with two different chemotherapy programmes and three patients (16.6%) with more than two programmes. Ten patients had had radiotherapy.

The most common tumour types were breast cancer (five patients) and soft tissue sarcoma (five patients). There was one patient each with epidermoid carcinoma of the cervix, multiple myeloma, gastric adenocarcinoma, and ganglioneuroblastoma. Seven patients – three complete response (CR) and four partial response (PR) – responded to previous treatment, while 11 patients had never presented an objective remission – seven stable disease (SD) and four progressive disease (PD).

Table II Characteristics of the patients

| | |
|---|---|
| Total number of patients | 18 |
| Age | |
| Median | 48 |
| Range | 20-63 |
| Gender (male-female) | 7:11 |
| Karnofsky | |
| Median | 70 |
| Range | 50-90 |
| Previous chemotherapy | 18 |
| Previous MDR-related drugs | 17 |
| Median number of MDR-related drugs | 2 |
| Range | 0-4 |
| Tumour type | |
| Breast carcinoma | 5 |
| Soft tissue sarcoma | 5 |
| Ewing's sarcoma | 2 |
| Non-small-cell lung carcinoma | 2 |
| Others | 4 |

Table III Haematological toxicity (number of cycles)[a]

| | *WHO toxicity grades* | | | |
|---|---|---|---|---|
| | *Leucocytes* | | *Platelets* | |
| *Dose level* | *III* | *IV* | *III* | *IV* |
| 10 mg $kg^{-1}$ $day^{-1}$ | – | 1 | 1 | 1 |
| 12 mg $kg^{-1}$ $day^{-1}$ | – | 1 | – | 1 |
| 14 mg $kg^{-1}$ $day^{-1}$ | 4 | 3 | – | 3 |
| 16 mg $kg^{-1}$ $day^{-1}$ | 4 | 4 | 1 | 2 |
| 18 mg $kg^{-1}$ $day^{-1}$ | 1 | – | – | – |

[a]Total number of cycles was 39.

Table IV Hyperbilirubinaemia and CsA dose level (number of cycles)

| | *WHO toxicity grades* | | | |
|---|---|---|---|---|
| *Dose level* | *I* | *II* | *III* | *IV* |
| 10 mg $kg^{-1}$ $day^{-1}$ | – | 2 | – | – |
| 12 mg $kg^{-1}$ $day^{-1}$ | 2 | – | – | – |
| 14 mg $kg^{-1}$ $day^{-1}$ | 5 | 3 | 1 | – |
| 16 mg $kg^{-1}$ $day^{-1}$ | 2 | – | – | – |
| 18 mg $kg^{-1}$ $day^{-1}$ | 1 | – | 4 | 1 |

### *Toxicity*

A total of 39 cycles were administered to the 18 patients (Table I). The median number of treatment cycles per patient was two, range 1–6. All treatment cycles were evaluated for toxicity. Reasons for withdrawal from treatment in the eight patients who received a single course of therapy were PD (six) and refusal of further therapy in presence of SD (two). Haematological toxicity was greater than expected for the same chemotherapy alone. Out of 39 cycles administered, 18 (46%) presented leucopenia grades III and IV, and nine (23%) had thrombopenia grades III and IV (Table III). Eleven out of 18 patients (61%) experienced leuco- or thrombopenia grades III and IV. One patient died of intracranial haemorrhage due to severe thrombopenia. Infectious episodes occurred in two patients. Of ten patients who received more than one treatment cycle, DOX and IFX were reduced in four (40%) owing to severe leucopenia and/or thrombopenia. Vomiting grades II and III occurred in 30 cycles but were well controlled with antiemetics and ceased rapidly after finishing the treatment cycles. Grade II mucositis occurred in five cycles and grade III in one. Four patients (22%) experienced this toxicity. Nephrotoxicity developed in eight cycles (20%), and consisted only of mild and reversible increases in blood creatinine. All patients who experienced this toxicity were treated at CsA c.i. doses of 14 mg $kg^{-1}$ $day^{-1}$ or greater. None of the patients presented severe nephrotoxicity. Mild and reversible hypomagnesaemia was seen in 28 out of 39 cycles (72%), but was well controlled with intravenous magnesium supplements. All patients experienced $>5\%$ and $<10\%$ increase in body weight during therapy due to fluid retention, which responded well to diuretics.

Reversible and asymptomatic hyperbilirubinaemia without alterations in hepatic enzymes, appeared in 18 out of 39 cycles (46%). The increase in total bilirubin was generally detected within the first 24 h of CsA infusion and was related to the dose of CsA (Table IV). Both direct and indirect bilirubin levels were increased, but direct bilirubin predominated. Hyperbilirubinaemia was rapidly reversible at the end of the treatment cycle, having lasted only 1 or 2 days. Mild reversible arterial hypertension was seen in six cycles (15%). The maximum registered systolic pressure was 170, the diastolic 100 mmHg. Anti-hypertensive medication was not required. Headache occurred in six cycles (15%) and was well controlled with non-opioid analgesics. In two patients with a less satisfactory control oral propranolol was successfully administered. This symptom was reversible after finishing treatment. Four patients presented pain in their previously known metastatic sites following the CsA loading dose, and sometimes required opioid analgesics to control it. Pain ceased soon after ending the cycle. There was no relationship between tumour response and appearance of pain in metastases. Only one of these four patients experienced symptomatic improvement. Anaphylactic reactions were not seen, although facial flushing occasionally occurred. No relevant neurological toxicity was observed. One patient with metastatic soft tissue sarcoma developed congestive cardiac failure grade II of the New York Heart Association (NYHA) after receiving a total cumulative DOX dose of 555 mg $m^{-2}$.

Table V CsA pharmacokinetics

| | *CsA dose level (mg $kg^{-1}$ $day^{-1}$)* | | | | |
|---|---|---|---|---|---|
| | *10* | *12* | *14* | *16* | *18* |
| Cycles assessed | 3 | 9 | 15 | 8 | 4 |
| Steady state (ng $ml^{-1}$) | 941.46 ± 68.7 | 1588 ± 292 | 1813 ± 507 | 2162 ± 467 | 2293 ± 807 |

### *CsA pharmacokinetics*

After the CsA loading dose (5 mg $kg^{-1}$) mean whole blood concentration was 2652 ng $ml^{-1}$. In Table V the mean steady-state CsA concentration values are shown. CsA blood levels showed a high interpatient variability. Whole blood levels of 1200 ng $ml^{-1}$ or greater were obtained at the second or higher dose level. Mean steady-state concentrations of 2000 ng $ml^{-1}$ were achieved at the fourth dose level (16 mg $kg^{-1}$ $day^{-1}$ CsA c.i.). Three more patients who received four treatment cycles were included in a fifth dose level at 18 mg $kg^{-1}$ $day^{-1}$. The mean steady-state CsA concentration at this level was 2293 ng $ml^{-1}$. Patient 10, a 44-year-old woman with breast cancer treated at the third dose level (14 mg $kg^{-1}$ $day^{-1}$ CsA c.i.), achieved CsA concentration of 1020 ng $ml^{-1}$ which was confirmed by repetition. This individual's low level as compared with her escalation group (see Table V) might be associated with her being treated chronically with phenobarbital to avoid seizures. This patient (the single one with brain metastases in our study) presented two small, well circumscribed and asymptomatic brain metastases as evidenced by MRI. After her first cycle she was treated at a higher dose level of 16 mg $kg^{-1}$ $day^{-1}$ CsA c.i. owing to the improvement of her peripheral disease (skin metastases), in order to see whether a higher CsA level could improve her brain lesions as well. Patient 16, a 21-year-old female with Ewing's sarcoma, who had received a single treatment course at 18 mg $kg^{-1}$ $day^{-1}$ CsA c.i., achieved a CsA blood concentration of 1291 ng $ml^{-1}$, which was in the range ef concentrations obtained with the second dose level (see Table V). The age of this patient may have contributed to the low CsA level obtained. Prior studies have shown that young adults may need higher doses to achieve a targeted CsA level (Yee *et al.*, 1986; Kahan and Grevel, 1988).

*Maximum tolerated dose and dose recommendations*

The MTD as defined in this study, was not reached. Dose escalations occurred from 10 to $18\,mg\,kg^{-1}\,day^{-1}$ CsA c.i. The single grade III toxicity observed, mucositis (excluding myelosuppression and nausea and vomiting), was seen in one patient treated at the third dose level. No other patient at this dose level experienced grade III or IV toxicity and further dose escalation continued. Hyperbilirubinaemia was not considered a dose-limiting toxicity since it was reversible, and no simultaneous alterations of liver enzymes were detected.

The objective of this study, to obtain a CsA plasma concentration of $2000\,ng\,ml^{-1}$ was achieved at the highest CsA doses. A loading i.v. CsA dose of $5\,mg\,kg^{-1}$ followed by a 3 day CsA c.i. at doses of 16 or $18\,mg\,kg^{-1}\,day^{-1}$, lead to a mean steady-state CsA concentration of $2000\,ng\,ml^{-1}$ or greater. At such levels toxicity is tolerable and reversible.

*T cell function studies (Figure 1)*

Comparing the different lymphocyte subpopulations seen before and 12 h after every cycle in 14 patients, there were significant decreases in the total lymphocyte counts ($P=0.0002$), and in CD19 ($P=0.0007$), CD3 ($P=0.0018$), CD4 ($P=0.0164$) and CD8 ($P=0.0003$) subpopulations. In seven patients who received more than one cycle, basal pretherapy counts were compared in the first and last treatments, with no significant differences in total lymphocyte counts ($P=0.8$), nor in CD19 ($P=0.7$), CD3 ($P=0.8$), CD4 ($P=0.3$) and CD8 ($P=0.8$) subpopulations. Similarly a significant increase in the CD4 CD8 lymphocyte ratio was detected in 14 patients ($P=0.02$), but again no differences were observed between the first and the last cycle of therapy in seven patients with more than one cycle ($P=1.0$). In 12 patients, PHA LTI showed no significant differences between basal and post-treatment values ($P=0.75$). In five patients who received more than one cycle, the value of basal LTI before the first cycle was similar to the value obtained before the last cycle ($P=0.50$).

*Responses*

There were two objective remissions (11%)(95% confidence intervals 1.38–34.71%). One patient (treated with CsA c.i. at $16\,mg\,kg^{-1}\,day^{-1}$) with an epidermoid carcinoma of the cervix with pulmonary and bone metastases, had a PR lasting 2.5 months and another patient (treated with CsA c.i. at $14\,mg\,kg^{-1}\,day^{-1}$) with metastatic Ewing's sarcoma, had a PR lasting 8 months.

Curiously, two patients (one with breast cancer and another with soft tissue sarcoma) who experienced PD showed a decrease of more than 50% in the size of isolated metastatic lesions with simultaneous increase of other lesions.

Finally, another patient (treated with CsA c.i. at $14\,mg\,kg^{-1}\,day^{-1}$) with metastatic breast cancer showed symptomatic pain relief and improvement in performance status without objective response in measurable tumour for 6 months.

### Discussion

Preliminary studies (Rodenburg *et al.*, 1991; Verweij *et al.*, 1991) using CsA as a modifier of drug resistance in combination with MDR cytotoxins in colon and renal carcinomas, showed that CsA levels of $1000-2000\,ng\,ml^{-1}$ could be achieved in the clinic with acceptable toxicity, although no activity was observed using this approach in such tumour types. These tumours are also resistant to many anti-cancer drugs which are not MDR related, including ifosfamide. Owing to the negative results of these trials and the intrinsic

**Figure 1** Changes in lymphocyte subsets. ■, before chemotherapy; □, after chemotherapy.

chemoresistance showed by such tumours, we decided to exclude tumours that constitutively express P-gp. In order to explore whether the modulating properties of CsA *in vitro* are of clinical relevance in resistant solid tumours, we undertook a phase I pilot study with intermittent high doses c.i. of CsA plus DOX and IFX. Besides its anti-tumoural effect, IFX was added because it causes glutathione depletion *in vitro* and *in vivo*, and may reverse DOX resistance. In fact, 72% of our patients were clinically resistant to DOX and an additional 11% to DHAD. In addition, 58% of the patients had failed to respond to prior combination chemotherapy that contained IFX.

The major aim of this study was to establish the dose of CsA needed to achieve a steady-state concentration of $2000\,ng\,ml^{-1}$ and the toxicity associated with this dose. Although we observed a great interpatient variability in the steady-state levels achieved, this study shows that a concentration of $2000\,ng\,ml^{-1}$ can be achieved with a CsA loading dose of $5\,mg\,kg^{-1}$ followed by a 3 day c.i. at doses $>$ $14\,mg\,kg^{-1}\,day^{-1}$ with acceptable toxicity.

Analysis of DOX pharmacokinetics was not an objective of our clinically oriented pilot study, since this question has been analysed by others, who found that a pharmacokinetic interaction between high doses of CsA and DOX really does exist (Sonneveld *et al.*, 1992; Bartlett *et al.*, 1993; Erlichman *et al.*, 1993). Although our results do not allow conclusions on possible pharmacological interactions, we have observed an increased myelosuppression with our protocol. We correlated our results with those of Millward *et al.* (1990), who used the same drugs at a similar planned dose intensity without CsA, but obtained a lesser haematological toxicity. Further evidence for greater haematological toxicity comes from our previous experience with similar doses of DOX and IFX in the treatment of metastatic soft tissue sarcomas (González-Manzano *et al.*, 1993). It is interesting to note the distribution of the haematological toxicity in our study. Most of the cycles (18 39 leucopenia and 9/39 thrombopenia) in which haematological toxicity was present, resulted in grade III or IV toxicity, and only one presented grade II leucopenia. This difference may be related to the pharmacokinetics of CsA, as shown by the high inter-patient variability observed in the steady-state CsA levels achieved.

Chronic treatments with CsA alone in patients receiving organ transplantation usually induce modifications in T-cell subsets, such as a decrease in the number of CD4 cells and, to a lesser extent, in the CD8 cells, with a concomitant decrease in the CD4 CD8 ratio (Awni, 1992). In such situations, CsA also decreases the LTI (Awni, 1992). Prior studies using intermittent high doses of CsA as a modifier of drug resistance in combination with chemotherapy, have suggested that a significant immunosuppression in patients receiving such treatments does not occur, but so far this has not been substantiated analytically. To our knowledge our present study is the first detailed report analysing the changes of several lymphocyte subsets and the LTI associated with intermittent high doses of CsA and chemotherapy. We have shown that short-term variations (a significant decrease in the CD3, CD4, CD8 and CD19 subpopulation counts) really occur early after treatment, and that these changes are reversible. This is shown in the patients who received more than one treatment cycle and yet did not show a significant decrease in the lymphocyte subpopulations studied. The LTI was not significantly modified by this treatment, either early after treatment or later. In addition, no opportunistic infections were observed. Taken together these results appear to suggest that this treatment can be safely administered without inducing a significant immunosuppressive status.

In contrast with the tendency of CsA and different schedules of combination chemotherapy to reduce the number of CD4 cells to a greater extent than CD8 cells, resulting in a decrease of the CD4/CD8 ratio (Olsen *et al.*, 1988; Favrot *et al.*, 1983; Linch *et al.*, 1983; Onsrud *et al.*, 1986), we have documented a significant increase of the CD4/CD8 ratio early after treatment with this protocol. We do not know whether such smaller CD4 decreases in comparison with CD8 cells occurring early after treatment may be considered a result of an effective modulation of the P-gp expressed by these lymphocyte subpopulations. Recent studies have evaluated the expression and activity of P-gp in human subpopulations of normal peripheral blood and bone marrow cells (Chaudhary *et al.*, 1992; Drach *et al.*, 1992). A functional P-gp was found to be expressed in all peripheral blood subpopulations (CD4, CD8, CD14, CD19, CD56) except granulocytes. In the study of Chaudhary *et al.* (1992), P-gp was expressed in the majority of the CD8, CD56 and CD20 peripheral blood cells (60–90%), but only in less than one-half of CD4 cells. Although an effective modulation of DOX by high doses of CsA explains our observations regarding the increase in the CD4/CD8 ratio, alternative explanations may be greater CD8 cell sensitivity to chemotherapy (than CD4 cells) and/or that CD8 cells have shorter survival times. The toxicities observed with our protocol were similar to those reported in other phase I trials with intermittent high doses of CsA given as modifier of drug resistance (Yahanda *et al.*, 1992; Erlichman *et al.*, 1993; List *et al.*, 1993). Such toxicities were: hyperbilirubinaemia (46% cycles), hypomagnesaemia (72% of cycles), hypertension (15%), nephrotoxicity (20%) and mild to moderate fluid retention (100%). Hypomagnesaemia was easily manageable and no clinical symptomatology was seen. Nephrotoxicity was mild and reversible in five patients. Three of them had received prior treatment with cisplatin, and a possible predisposition to experience nephrotoxicity in these patients cannot be discarded. Nevertheless, we have observed a similar incidence of this toxicity as compared with other recently published series (Yahanda *et al.*, 1992; Erlichman *et al.*, 1993), and no episodes of acute renal failure have been observed. Two patients obtained a PR with our protocol. Interestingly, one of them was a patient with Ewing's sarcoma who was progressing after treatment with the same drugs without CsA. The addition of CsA to the same agents that failed previously induced a PR. This patient presented acquired resistance to prior DOX-based chemotherapy.

It remains to be elaborated whether adequate levels of CsA reached the tumour cells, in our study as well as past studies. The inaccessability of metastatic lesions in most of our cases precluded tumour biopsy for determination of CsA. At present, it is not known whether adequate plasma levels of CsA are associated with good intratumour levels. It would be convenient to address such question in the design of future studies with CsA, although feasibility may constitute a significant problem in solid tumours.

In summary, CsA steady-state levels of 2000 ng $ml^{-1}$ can be achieved with a loading dose of 5 mg $kg^{-1}$ followed by a 3 day CsA c.i. at a dose of 16 mg $kg^{-1}$ $day^{-1}$ in combination with DOX and IFX, with acceptable toxicity. Although some encouraging responses have been observed, and an attempt to modulate resistance by different mechanisms appears interesting, further evaluation is needed to establish the clinical value of this approach in selected tumour types.

### References

AWNI WM. (1992). Pharmacodynamic monitoring of cyclosporin. *Clin. Pharmacokinet.*, **23** (6): 428–448.

BARTLETT NL, FISHER GA, HALSEY J, EHSAN MN, LUM BL AND SIKIC BI. (1993). A phase I trial of doxorubicin (D) with cyclosporine (CsA) as a modulator of a multidrug resistance (MDR). *Proc. Am. Soc. Clin. Oncol.*, **12**, A366.

CAMPOS L, GUYOTAT D, ARCHIMBAUD E, CALMARD OP, TSURUO T, TRONCY J, TREILLE D AND FIERE D. (1992). Clinical significance of multidrug resistance P-glycoprotein expression on acute nonlymphocytic leukemia cells at diagnosis. *Blood*, **79**, 473–476.

CHAN HS, THORNER P, HADDAD G AND LING V. (1990). Immunohistochemical detection of P-glycoprotein: Prognostic correlation in soft tissue sarcoma of childhood. *J. Clin. Oncol.*, **8**, 689–704.

CHAN HS, HADDAD G, THORNER P, DE BOER G, LIN YP, ONDRUSEK KN, YEGER H AND LING V. (1991). P-glycoprotein expression as a predictor of the outcome of therapy for neuroblastoma. *N. Engl. J. Med.*, **325**, 1608–1614.

CHAUDHARY PM, MECHETNER EB AND RONINSON IB. (1992). Expression and activity of the multidrug resistance P-glycoprotein in human peripheral blood lymphocytes. *Blood*, **80**, 2735–2739.

DRACH D, ZHAO S, DRACH J, MAHADEVIA R, GATTRINGER C, HUBER H AND ANDREEF M. (1992). Subpopulations of normal peripheral blood and bone marrow cells express a functional multidrug resistant phenotype. *Blood*, **80**, 2729–2734.

ERLICHMAN C, MOORE M, THIESSEN JJ, KERR IG, WALKER S, GOODMAN P, BJARNASON G, DE ANGELIS C AND BUNTING P. (1993). Phase I pharmacokinetic study of cyclosporin A combined with doxorubicin. *Cancer Res.*, **53**, 4837–4842.

FAVROT M, JANOSSY G, TIDMAN N, BLACKLOCK H, LOPEZ E, BOFILL M, LAMPERT I, MORGENSTEIN G, POWLES R, PRENTICE HG AND HOFFBRAND AV. (1983). T cell regeneration after allogeneic bone marrow transplantation. *Clin. Exp. Immunol.*, **54**, 59–72.

GOLDSTEIN LJ, GALSKI H, FOJO A, WILLINGHAM M, LAI SL, GAZDAR A, PIRKER R, GREEN A, CRIST W, BRODEUR GM, LIEBER M, COSSMAN J, GOTTESMAN M AND PASTAN I. (1989). Expression of a multidrug resistance gene in human cancers. *J. Natl. Cancer Inst.*, **81**, 116–124.

GONZÁLEZ-MANZANO R, VIEITEZ JM, TANGCO E, DE ALAVA E, HERRANZ P AND GARCÍA-FONCILLAS J. (1993). Phase II evaluation of doxorubicin, ifosfamide, and dacarbazine plus amphotericin B in the treatment of metastatic soft tissue sarcomas: a pilot study. *Am. J. Clin. Oncol.*, **16**, 332–337.

KAHAN BD AND GREVEL J. (1988). Optimization of cyclosporine therapy in renal transplantation by a pharmacokinetic strategy. *Transplantation*, **46**, 631–644.

KASHAMI-SABET M, WANG W AND SCANLON KJ. (1990). Cyclosporin A suppresses cisplatin-induced c-fos gene expression in ovarian carcinoma cells. *J. Biol. Chem.*, **265**, 11285–11288.

LARSSON R AND MYGREN P. (1990). Verapamil and cyclosporin A potentiate the effects of chemotherapeutics agents in the human medullary thyroid carcinoma TT line not expressing the 170 Kda P-glycoprotein. *Cancer Lett.*, **54**, 125–131.

LEE FYF, VESSEY AR AND SIEMANN DW. (1988). Glutathione as a determinant of cellular response to doxorubicin. *Natl Cancer Inst. Monogr.*, **6**, 211–215.

LINCH DC, KNOTT LJ, THOMAS RM, HARPER TP, GOLDSTONE AH, DAVIS EG AND LEVINSKI RJ. (1983). T cell regeneration after allogeneic and autologous bone marrow transplantation. *Br. J. Haematol.*, **53**, 451–458.

LIND MJ, MCGOWN AT, HADFIELD JA, THATCHER N, CROWTHER D AND FOX BW. (1989). The effect of ifosfamide and its metabolites on intracellular glutathione levels in vitro and in vivo. *Biochem. Pharmacol.*, **38**, 1835–1840.

LIST AF, SPIER C, GREER J, WOLFF S, HUTTER J, DORR R, SALMON S, FUTSCHER B, BAIER M AND DALTON W. (1993). Phase I/II trial of cyclosporine as a chemotherapy resistance modifier in acute leukemia. *J. Clin. Oncol.*, **11**, 1652–1660.

MARIE JP, ZITTOUN R AND SIKIC BI. (1991). Multidrug resistance (mdr1) gene expression in adult acute leukemias: Correlations with treatment outcome and in vitro drug sensitivity. *Blood*, **78**, 586–592.

MILLWARD MJ, HARRIS AL AND CANTWELL BMJ. (1990). Phase II study of doxorubicin plus ifosfamide/mesna in patients with advanced breast cancer. *Cancer*, **65**, 2421–2425.

OLSEN GA, GOCKERMAN JP, BAST RC, BOROWITZ M AND PETERS WP. (1988). Altered immunologic reconstitution after standard-dose chemotherapy or high-dose chemotherapy with autologous bone marrow support. *Transplantation*, **46**, 57–60.

ONSRUD M, BOSNES V AND GRAHM I. (1986). Cis-platinum as adjunctive to surgery in early stage ovarian carcinoma: effects on lymphoid cell subpopulations. *Gynecol. Oncol.*, **23**, 323–328.

PASTAN I AND GOTTESMAN MM. (1991). Multidrug resistance. *Annu. Rev. Med.*, **42**, 277–286.

PIRKER R, WALLNER J, GEISSLER K, LINKESCH W, HAAS OA, BETTELHEIM P, HOPFNER M, SCHERRER R, VALENT P, HAVELEC L, LUDWIG H AND LECHNER K. (1991). MDR1 gene expression and treatment outcome in acute myeloid leukemia. *J. Natl Cancer Inst.*, **83**, 708–712.

RODENBURG CJ, NOOTER K, HERWEIJER H, SEYNAEVE C, OOSTEROM R, STOTERG G AND VERWEIJ J. (1991). Phase II study of combining vinblastine and cyclosporin-A to circumvent multidrug resistance in renal cell cancer. *Ann. Oncol.*, **2**, 305–306.

SONNEVELD P, DURIE BGM, LOKHORST HM, MARIE JP, SOLBU G, SUCIU S, ZITTOUN R, LOWENBERG B AND NOOTER K. (1992). Modulation of multidrug-resistant multiple myeloma by cyclosporin. *Lancet*, **340**, 255–259.

TWENTYMAN PR, FOX NE AND WHITE DJG. (1987). Cyclosporin A and its analogues as modifiers of adriamycin and vincristine resistance in a multidrug resistant human lung cancer cell line. *Br. J. Cancer*, **56**, 55–57.

VERWEIJ J, HERWEIJER H, OOSTEROM R, VAN DER BURG MEL, PLANTIN AST, SEYNAEVE C, STOTER G AND NOOTER K. (1991). A phase II study of epidoxorubicin in colorectal cancer and the use of cyclosporin-A in an attempt to reverse multidrug resistance. *Br. J. Cancer*, **64**, 361–364.

WORLD HEALTH ORGANIZATION. (1979). *Handbook for Reporting Results of Cancer Treatment*, Offset publication 48. World Health Organization: Geneva.

YAHANDA AM, ADLER KM, FISHER GA, BROPHY NA, HALSEY J, HARDY RI, GOSLAND MP, LUM BL AND SIKIC BI. (1992). Phase I trial of etoposide with cyclosporine as a modulator of multidrug resistance. *J. Clin. Oncol.*, **10**, 1624–1634.

YEE GC, LENNON TP, GNUR DJ, KENNEDY MS AND DEEG HJ. (1986). Age-dependent cyclosporine: Pharmacokinetics in marrow transplant recipients. *Clin. Pharmacol. Ther.*, **40**, 438–443.